# Application of Genome Editing in Tomato Breeding: Mechanisms, Advances, and Prospects

**DOI:** 10.3390/ijms22020682

**Published:** 2021-01-12

**Authors:** Hymavathi Salava, Sravankumar Thula, Vijee Mohan, Rahul Kumar, Fatemeh Maghuly

**Affiliations:** 1 Department of Plant Sciences, University of Hyderabad, Hyderabad 500064, India; hyma.salava56@gmail.com; 2 Mendel Centre for Plant Genomics and Proteomics, Central European Institute of Technology (CEITEC), Masaryk University, Kamenice 5, CZ-625 00 Brno, Czech Republic; sravankumar.thula@ceitec.muni.cz; 3 Department of Biological Sciences, University of North Texas, Denton, TX 76203, USA; Vijee.Mohan@unt.edu; 4 Plant Translational Research Laboratory, Department of Plant Sciences, University of Hyderabad, Hyderabad 500064, India; rksl@uohyd.ac.in; 5 Plant Functional Genomics, Institute of Molecular Biotechnology, Department of Biotechnology, BOKU-VIBT, University of Natural Resources and Life Sciences, 1190 Vienna, Austria

**Keywords:** trait improvement, gene knockout, resistance breeding, biotic stress, abiotic stress

## Abstract

Plants regularly face the changing climatic conditions that cause biotic and abiotic stress responses. The abiotic stresses are the primary constraints affecting crop yield and nutritional quality in many crop plants. The advances in genome sequencing and high-throughput approaches have enabled the researchers to use genome editing tools for the functional characterization of many genes useful for crop improvement. The present review focuses on the genome editing tools for improving many traits such as disease resistance, abiotic stress tolerance, yield, quality, and nutritional aspects of tomato. Many candidate genes conferring tolerance to abiotic stresses such as heat, cold, drought, and salinity stress have been successfully manipulated by gene modification and editing techniques such as RNA interference, insertional mutagenesis, and clustered regularly interspaced short palindromic repeat (CRISPR/Cas9). In this regard, the genome editing tools such as CRISPR/Cas9, which is a fast and efficient technology that can be exploited to explore the genetic resources for the improvement of tomato and other crop plants in terms of stress tolerance and nutritional quality. The review presents examples of gene editing responsible for conferring both biotic and abiotic stresses in tomato simultaneously. The literature on using this powerful technology to improve fruit quality, yield, and nutritional aspects in tomato is highlighted. Finally, the prospects and challenges of genome editing, public and political acceptance in tomato are discussed.

## 1. Introduction

Tomato (*Solanum lycopersicum*) is an economically important crop with almost 160 million tons produced in 2016 (FAO, 2016). Tomato is a diploid plant with 12 chromosomes and a genome size of ~950 Mb. It is one of the most important horticultural crops worldwide because of its nutritional value and derived industrial products. It is also an optimal bridge between the model plant (e.g., Arabidopsis) and other crops due to the availability of enormous genetic and genomic resources. Tomato and its 12 wild relatives are native to western and central South America and are widespread throughout diverse habitats, contributing to high genetic variability. Little is known about the history of tomato domestication [1], but it was at an advanced level before it reached the old world. During the domestication of tomatoes, intense levels of improvement occurred worldwide. Consequently, many morphologically distinct cultivars have been developed from the single species of *S. lycopersicum*. Unfortunately, some important traits, such as resistance to biotic and abiotic factors, which existed in the wild tomatoes, have been compromised during the domestication. As a result, conventional breeding has resulted in improved traits accompanied by loss of fitness and genetic diversity. However, it is a highly time-consuming and laborious task due to backcrosses [2]. Technological advancement in genomics improved the genetic engineering of crops in the last two decades. The genetic engineering also referred to as recombinant DNA technology involves the transfer of desired gene from one species to another, thereby broadening the chances for crop improvement [3]. The transgenic plants developed using this technology are named as genetically modified organisms (GMOs). However, the regulatory approval of GMOs is a major drawback as the release of GM crops to the public market is costly and often delayed [4].

Recent efforts have ensured the conservation of landraces and wild species that allowed re-introducing resistance traits [5,6]. Besides, artificial mutagenesis offers breeders with genotypes containing novel genetic and phenotypic diversity that helps in enlarging the narrowed genetic base [7,8,9]. Mutant collections also provide a complementary alternative for trait discovery in tomato, providing an allelic series in a uniform genetic background [10]. In the last decade, various tomato cultivars were used for generating mutant collections [8,9,11,12,13,14,15,16,17,18,19,20,21,22,23].

Modern genetics and breeding methods have contributed to the understanding and developing of structural and functional aspects of tomato genomes [10]. In tomato, quantitative trait loci (QTL) mapping has assisted in mapping of genes associated with heat and salt tolerance [24,25,26] and various fruit-related traits [27,28,29]. Further, genome-wide association study (GWAS) has been used to map loci related to traits such as plant architecture, fruit shape, and fruit weight in tomato [30,31,32], and fruit metabolites [33,34,35]. GWAS also helped identify loci related to drought and salt tolerance in tomato [36].

With the advancement in high-throughput sequencing technologies, genomes of tomato [37,38,39,40] have been fully sequenced, including several wild tomato species and landraces [39,41,42,43,44]. The information on genomic sequences of wild tomato cultivars and other tomato accessions are available in Sol Genomics Network (https://solgenomics.net/) and *Solanum pennellii* genome project (https://www.plabipd.de/project_spenn/start.ep). The availability of a high-quality genome sequence and Rapid improvement in molecular biology and genomics techniques enabled researchers to precisely edit any desired genomics locus in the form of insertions/deletions or base substitution. The availability of pan-genome could facilitate gene-editing tools to test the effect of target gene modifications in tomato breeding and development. 

Here we review the structure, mechanism of various genome editing tools, and genome editing approaches for major breeding goals in tomato, such as resistance to various biotic and abiotic stresses and traits improvement. We also would like to shed some light on future applications in the field concerning GMOs public and political acceptance. 

## 2. Structure and Mechanism of Genome Editing Tools

Diversification of organisms is based on variations in the genetic pool [45]. Genetic variation is the basis for improving an organism’s traits and is valuable for the production of new cultivars in plant breeding [46]. During evolution, genetic variations occur spontaneously due to DNA damage or errors in the replication process, which are termed as mutations. This process is called mutagenesis [47,48]. The natural mutations are spontaneous, sporadic, and random; therefore, it is impossible to rely only on natural variations for crop breeding [49,50]. Thus, artificial mutagenesis is needed to increase genetic variation, an essential step for the breeding program [49]. 

Genome editing (GE) techniques have revolutionized the biological world by facilitating precise, efficient, and targeted modification at genomic loci of the living organisms, including microbes, animals, humans, and plants. The primary mechanism of GE includes double-stranded breaks in the DNA (dsDNA) by specific engineered nucleases. The dsDNA break is repaired, either by non-homologous end joining (NHEJ) or homology-dependent recombination (HDR). HDR allows generating accurate point mutations, deletions, or gene knock-in useful for crop breeding but with extremely low editing frequencies. In contrast, NHEJ is error-prone and allows random small insertions or deletions as well as substitutions, preferably causing a gene knockout [51]. 

**Figure 1:**
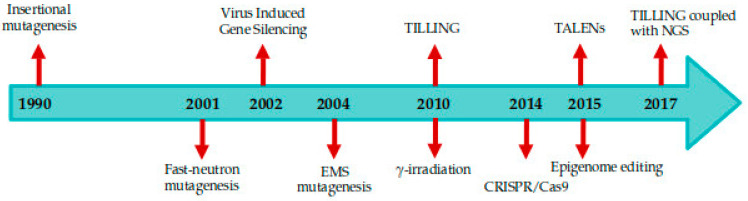
Timeline of the breakthrough of genome editing in tomato. Insertional mutagenesis [55], Virus Induced Gene Silencing (VIGS) [56], Targeted Induced Local Lesions In Genomes (TILLING) [15], TALENs [57], TILLING coupled with NGS [58], Fast-neutron mutagenesis [59], ethyl methane sulphonate (EMS) mutagenesis [9], γ-irradiation [60], CRISPR/Cas9 [61], Epigenome editing [62].

Several genome editing approaches are available for inducing site-specific dsDNA breaks such as induced mutagenesis, Oligonucleotide directed mutagenesis (ODM), epigenome editing, transposable elements, Zinc finger nucleases (ZFNs), transcriptional activator like effector nucleases (TALENs), and more recently clustered regularly interspaced short palindromic repeat (CRISPR/Cas9) systems can be exploited to decipher the role of unannotated and uncharacterized genes [12,52,53,54]. The timeline of the genome editing tools is represented in Figure 1.

### 2.1. Induced Mutagenesis

**Figure 2:**
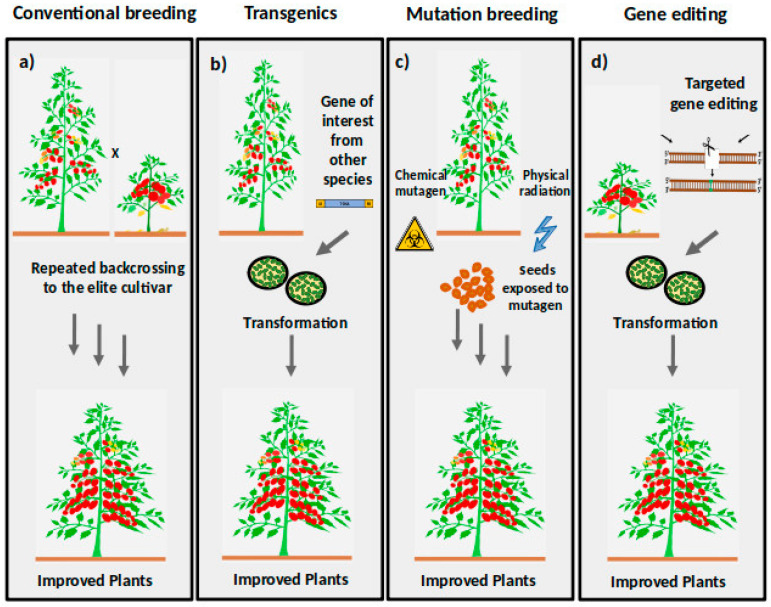
Represents the breeding techniques employed for crop improvement. (**a**) Conventional breeding involves selective breeding of two tomato species with desired traits. For example, a tall plant with low yield and stress tolerance is crossed to a small plant with bigger fruits. Repeated backcrossing is performed to the elite cultivar to generate plants with desired traits. Conventional breeding is mostly accompanied with loss of genetic diversity due tote selection process. (**b**) Transgenic breeding involves the introduction of a desired gene (transgene) from other species into the selected plant by the transformation process. (**c**) Mutation breeding involves physical radiation or a chemical mutagen to induce mutations. The mutated populations (M_1_) are generated, and to reduce chimerism M_2_ or higher populations are produced. The mutant is then screened either by forward or reverse genetics. (**d**) Targeted genome editing (detailed described in Section 2.7), schematic describes the procedure for generating a wide variety of improved plant traits. After generation of edited plants are then screened phenotypic and genotypic for discovering the plant with desirable traits.

Induced mutagenesis allows the introduction of novel genetic alleles and facilitates novel genetic resources for crop improvement and gene function discovery. Since the initial reports of mutation breeding by Stadler in 1928, remarkable progress has been accomplished in genetic breeding techniques [63]. It has been widely used in crop plants with low genetic variability and those species that are not amenable to conventional breeding (Figure 2a). The mutagenized population, once generated, becomes an everlasting resource. Due to their long safety record and absence of foreign DNA (Figure 2b), mutagenic plants are exempted from the EU GMO legislation. However, the generation of a mutant population is laborious and time-consuming. Based on the inducing agent, the mutagenesis is further classified into physical and chemical mutagenesis (Figure 2c). 

#### 2.1.1. Physical Mutagenesis

Since the 1920s, the physical mutagenic sources found to be effective for inducing mutations are ionizing radiations. The physical mutagens include ionizing (electromagnetic) radiations such as X-rays, cosmic rays, α-rays and β-rays, γ-rays, neutrons, and protons while non-ionizing source includes UV rays [64]. Even though physical mutagenesis has proved to be very effective particularly for producing large DNA fragment deletions, its application in inducing mutagenesis is mostly directed towards the generation of knockout mutants and rearrangement of genes [65]. X-rays and γ-rays are the most commonly used. Exposure of plants to the γ-radiation causes damage such as double-strand breaks and also produces a range of damage to DNA due to the production of free radicals. Fast-neutron mainly causes deletion mutations ranging from few base pairs to few kilo bases while γ-rays irradiation can cause large deletions along with chromosomal rearrangements [12,66,67].

In addition, ion beams such as protons, helium, and heavy charged particles are known to be highly mutagenic. They are accelerated at higher speeds with high linear energy transfer that induce larger DNA lesions [68,69]. These ion beams radiations induce single- and double-stranded breaks, which lead to inversions, deletions, point mutations, and translocations [68,70]. In Arabidopsis, various novel mutations were identified using high-speed carbon ions [67]. Although various sources of physical mutagenesis are available, majority of the mutants induced using physical mutagens were generated by γ-irradiation. 

There are over 3200 varieties of induced mutant lines available at the Food and Agriculture Organization of the United Nations and the International Atomic Energy Agency (Joint FAO/IAEA) in Vienna [71]. Several mutants are obtained using physical mutagenesis in various plant species such as Arabidopsis [72,73,74,75], rice [76,77], and tomato [9,14,60,78]. 

The tomato *tangerine* mutant (carotenoid isomerase, *CRTISO*) was identified from fast-neutron mutagenesis [79] by map-based cloning in Micro-Tom [14,59]. In a different study, 6301 mutant lines were generated using γ-ray irradiation in Micro-Tom with variable phenotypes such as fruit size, color, ripening, flower and leaf morphology, brix, etc., which provides a valuable genetic resource for breeding and functional genomics in tomato [78]. Another study generated 865 mutants by fast neutron and 2552 mutants induced by EMS in tomato cultivar M82 and traits like plant height, fruit size, fruit color, ripening, sterility, and plant stress response (for example, Leaf curl disease) were examined [9]. 

#### 2.1.2. Chemical Mutagenesis

The chemicals used for mutagenesis in plants include alkylating agents, purine analogs, oxidizing agents, sulphonic esters, and epoxides [80]. Chemical mutagenesis is advantageous over physical as it does not require sophisticated pieces of equipment. This mutagenesis induces point mutations or single base substitutions that often lead to gain or loss of functions giving rise to novel allelic mutants instead of large deletions or chromosomal rearrangements.

Different alkylating agents such as ethyl methane sulphonate (EMS), Diethyl sulfate (DES), ethyleneimine (EI), ethyl nitro urethane (ENU), 1-methyl-1-nitrosourea (MNU), ethyl nitrosourea (ENU), and azides are used for mutagenesis [81]. Among these, EMS is the most desired chemical mutagen in plants [9,15,82,83] that particularly alkylates guanine bases and transfers reactive alkyl groups to other molecules [81]. It predominantly induces point mutations randomly in the genome of the species with the majority being G/C to A/T base pair transitions [84]. Like EMS, MNU mainly induces G/C to A/T transitions and also induces translocations and inversions at lower frequencies [85,86]. In contrast to MNU, ENU significantly induces G/C to A/T transitions together with A/T to G/C transitions including transversions [87]. In addition, analogues of nitrogenous bases such as maleic hydrazide, 5-bromouracil, and 2-aminopurine also possess mutagenic activity. However, these bases are rarely used in plants [86].

EMS has been successfully exploited in tomato cultivars such as Moneymaker [88], M82 [9,89], *Lycopersicon esculentum* Mill. [90], and Red setter [7,15,91], Arka Vikas [58]. In tomato, the first mutant identified from EMS (60 mM) was *adh-1,* which encodes for alcohol dehyrogenase1 (*ADH1*) [88]. *Adh-1* is a biochemical mutant, which renders the likelihood of screening large populations as only the mutants survive in the presence of allyl alcohol [88]. In 2007, Kostovś group generated an EMS (1.5%) population in *L. esculentum Mill.* and identified 16 plants resistant to *Orabanche ramosa* and this mutant population can be used to study Broomrape resistance in tomato breeding [90]. In another study, 0.7% and 1% EMS was used in Red setter cultivar to produce 5508 lines and studied seven fruit quality traits to identify 66 point mutations. This mutant population was developed for various forward and reverse genetic screening [15]. The miniature dwarf tomato cultivar Micro-Tom was used to create EMS mutant population with two different doses (0.5% and 1%) [16,82], out of which 1% EMS was found to be efficient to generate mutant population to perform functional genomic studies in tomato [7]. 

To screen a mutation population for mutation detection within genes of interest forward genetics (correlate phenotype to gene) or reverse genetic (correlate gene to phenotype) can be used. Targeted induced local lesions in genomes (TILLING) is a high-throughput reverse genetic tool and a well-known approach to identify point mutations in specific genes in the mutagenized population and also to study gene function [15,16]. Three essential steps are necessary for optimal results in TILLING approach; first, the right pooling strategies, second, a good gene model and protein conservation model, which could help to select the gene region with the highest number of possible variations, and third, a suitable PCR primer pairs for a product of approximately 1000–2000 bp. Finally, identified mutations will be confirmed and evaluated by sequencing [92,93]. Ultimately, it can provide an allelic series of silent, mis-sense, non-sense, and splice site mutations to examine the effect of various mutations in a gene. TILLING has been used in Arabidopsis [94], maize [95], barley [96], wheat [97], tomato [15,16,58], lotus [98], etc. 

Currently, the conventional TILLING is being replaced by next-generation sequencing (NGS)-based mutation detection, as it allows rapid and accurate screening of a large number of amplicons in a short duration of time through sequencing of smaller amplicons (about 300 bp) [99,100,101,102]. In the first application of NGS in tomato, Rigola and group identified two novel *SlelF4E* alleles in using 3D pooling, named the approach as “Keypoint” technology [100]. In 2017, TILLING coupled with NGS technology was used to screen a tomato EMS population of 2300 lines and identified 64 mutations with a mutation frequency of 1 in 367 Kb [58].

#### 2.1.3. Limitation of Physical and Chemical Mutagenesis

Physical and chemical mutagenesis are random, and their mutations spectra are not well known. Besides, the optimal dose rate of each mutagen needs to be determined for each genotype. They are time and cost-intensive since they require large populations (5000–10,000 individuals) to select desired phenotype and high-throughput methods for screening mutation rate at the genetic level. Moreover, physical and chemical mutagenesis requires highly sophisticated equipment and infrastructure to ensure safety use, which can be afforded only in labs with specialized laboratory setup. Some physical mutagens such as γ-ray are highly radioactive, and chemical mutagens are hazardous [93,103].

### 2.2. Oligonucleotide Directed Mutagenesis (ODM)

ODM is known as site-directed mutagenesis or site-specific mutagenesis. It is also called gene targeting or directed gene modification and recently termed precision gene editing [104]. It is a method to introduce specific variations in the target gene of interest. The specific DNA changes include substitutions, insertions, and deletions. The ODM can induce mutations in a particular gene of interest, study the protein function as a result of alterations in the DNA, and introduce or remove sites of restriction enzymes [105]. 

This technique was first successfully illustrated in mammalian systems in 1996 [106,107], and later it was implemented by researchers in plants [108]. The primary mechanism of ODM came from research on prokaryotes and eukaryotes. ODM’s basic procedure involves the transport of oligonucleotide (that is complementary to the gene of interest) carrying a mutation into the cell via cell membrane and nuclear membrane. It reaches the nucleus where the oligonucleotide binds to the complementary DNA [108]. The host mismatch repair system corrects the DNA damage, and the mutations get incorporated into the genome, inducing a site-specific mutation.

Although ODM has been successfully executed in plants such as maize [109], tobacco [110], rice [111], and wheat [112] that are resistant to herbicides, ODM techniques have shown a relatively low correction rate [104]. However, there are no reports of ODM in tomato yet.

### 2.3. Epigenome Editing

Epigenetic gene regulation is another important aspect of gene regulation. DNA methylation is a conserved mechanism to regulate gene expression and repress transposon activity [113]. Epigenome editing or engineering refers to employing tools to induce epigenetic changes at a particular location on the genome. This editing is dependent on the methylation status and chromatin organization of the genome. De novo DNA methylation in plants is mediated by RNA-dependent DNA methylation (RdDM) pathway, which involves RNA pol IV and RNA pol V [114,115]. MET1 is the major methyltransferase along with chromomethylases (CMTs), and Domains rearranged methyltransferases (DRM), which maintain the CG/CHG/CHH cytosine methylation in the plant genome [116,117].

De novo DNA methylation in plants involves two sequential steps, biogenesis of short interfering RNAs (siRNAs) and targeting the methylated sequences [118]. The RdDM methylation occurs in two ways-canonical pathway (naturally occurring signaling pathway inside the living system) and non-canonical pathway (induced signaling pathway by chemicals or xenobiotics) [119]. In the canonical pathway, the RNA pol IV transcribes the heterochromatin to single-stranded RNA (ssRNA). The RNA-dependent RNA polymerase (RDR2) converts the ssRNA to transcribe into double standard RNA (dsRNA) [120,121]. The physical interaction of RNA pol IV and RDR2 generates 26–45 nucleotide fragments of dsRNA, which are further cleaved by Dicer-like3 (DCL3) to yield 24 nucleotides siRNAs [121,122]. The non-canonical pathway also exists but is not very common [119]. Several studies have been reported on epigenomic editing in various plant species such as Arabidopsis [123,124,125,126,127], tobacco [128], maize [129], potato [130], and rice [131].

Although the fruit epigenome and full complement of METs, CMTs, and DRMs are known in tomato, there are limited studies on epigenome editing in tomato. In tomato, the first evidence of DNA methylation status affecting fruit ripening came from the studies of colorless non-ripening (CNR), which encodes a *SQUAMOSA promoter binding protein3-like* (*SPB3-like*). The normal ripening is hindered in *cnr* epimutant producing colorless pericarp where *SPB3-like* gene is hypermethylated, repressing the fruit ripening transcription factors (TFs) and carotenoid biosynthesis [132]. The methylation status of the ripening associated genes is an important factor controlling the transition of the tomato fruit development [133]. Further, another group demonstrated that the DNA methylation in tomato is in turn regulated by DEMETER –like DNA methylases (DMLs) [62]. Knockdown of *SlDML2* inhibited the tomato fruit ripening by hypermethylation of the key TFs involved in fruit ripening such as RIN, CNR, and NOR [62]. AlkB homolog 2 (SlAlkBH2) binds to and stabilizes the transcripts of *SlDML2* by demethylation of 6-methyl adenosine. The CRISPR/Cas9 knockout of *SlAlkBH2* also resulted in reduced transcript levels of *SlDML2* and delay in fruit ripening [134]. AlkBH2 is α-ketoglutarate-dependent dioxygenase and repairs the alkylated bases in DNA and mRNA by oxidative demethylation [135]. However, there are limited studies on epigenetic changes governing stress conditions. Rossi and Iusem [136] were the first to show that Asr1 is induced by ABA, water stress, and ripening. *Asr1* stands for ABA, stress, and ripening [136]. Later, another gene called *Asr2* was identified to be stimulated in leaves and roots of tomato plants upon water stress [137]. Interestingly, *Asr2* promoter was demethylated at CNN sites upon exposure to water deficit stress [138]. The emerging high-throughput technologies would enable the discovery of epialleles required for stress tolerance in crop plants in the future.

#### RNA Interference (RNAi)

RNA silencing is the natural mechanism exploited by a diverse range of organisms such as protozoa, fungi, animals, and crop plants as a defense response to pathogens like viruses [139]. This phenomenon was first discovered in *Caenorhabditis elegans* in 1998 [140]. The gene silencing occurs in two ways—transcriptional gene silencing (TGS) and post-transcriptional gene silencing (PTGS) [141,142]. TGS represses mRNA through promoter methylation while in PTGS, dsRNA induces mRNA degradation [64,143,144,145]. In plants, there are several approaches to silence the target gene expression. Virus-induced gene silencing (VIGS) is once such a tool in PTGS for the functional characterization of genes in plants [146]. It can be used both as forward and reverse genetic tools in plants. Plants infected by virus uses the PTGS mechanism to induce dsRNA and destroy the viral RNAs as a defense response [147,148,149].

**Figure 3:**
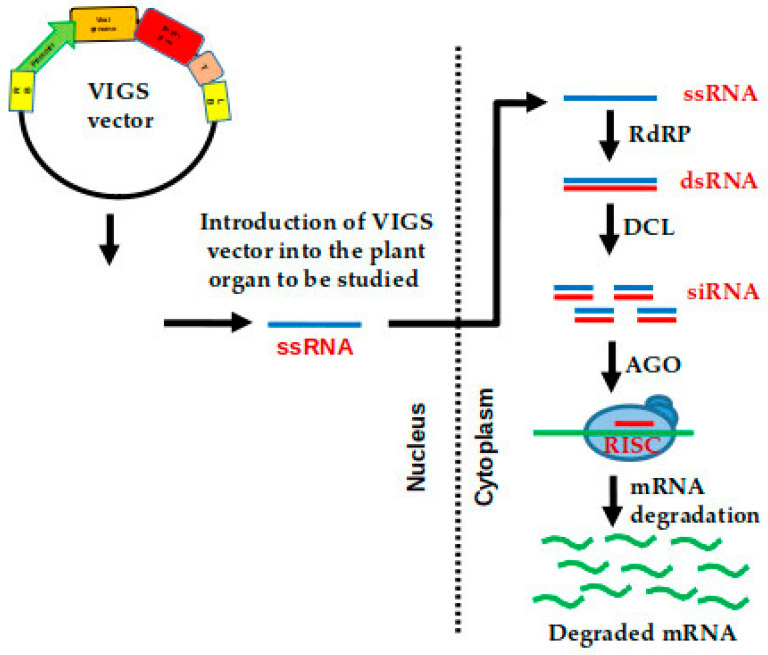
Represents the virus-induced gene silencing (VIGS) mechanism in tomato. This technique allows transient expression of the target gene introduced through viral vectors into the plant. Once the vector is released in the plant, the single-stranded RNA (ssRNA) is converted to double-stranded RNA (dsRNA), which is further cleaved by dicer-like (DCL) enzymes and loaded to protein Argonaute (AGO) to generate short interfering RNAs (siRNA). The siRNA enters the RNA-induced silencing complex (RISC) to initiate the degradation of targeted mRNA, thereby silencing the gene function. RNA dependent RNA polymerase (RdRP). VIGS vector contains: Left Border (LB) and Right Border (RB) highlighted in yellow; Promoter in green; Viral genome in orange; Target gene in red; Terminator (T).

The term VIGS was coined by Van Kammen in 1997 [150]. VIGS also follows the same mechanism as RNA-induced gene silencing, where dsRNAs are produced from the target gene by host RNA dependent RNA polymerase (RdRP). The DICER-like enzyme cleaves the dsRNA into short interfering RNA (siRNA) of 21–25 nucleotides in length with 2 nucleotides 3′ overhangs [151,152]. Subsequently, these siRNAs are introduced into the RNA-induced silencing complex (RISC) complex. This complex consequently targets the siRNA to the complementary RNA resulting in RNA degradation, abolishing the translation of the mRNA [153,154] (Figure 3).

*Tobacco Mosaic Virus* (TMV) was the first modified virus vector used for VIGS to suppress the expression of phytoene desaturase (*PDS*) in *Nicotiana benthamiana* [155]. Later, other viruses such as *Tobacco Rattle Virus* (TRV), *Turnip yellow Mosaic Virus* (TYMV), and *Potato virus X* (PVX) were modified for VIGS studies in plants. Modified TRV-based was used for efficient gene silencing in tobacco [156] and tomato [56]. TRV is advantageous over other VIGS vectors as it is easy to introduce TRV-based VIGS vector in plants, especially in Solanaceous plants [56]. Another advantage of using TRV is that TRV infection spreads more rapidly all over the plant. However, TRV symptoms are low [56,157]. *Potato virus X* (PVX) has a limited host range of three plant families, whereas only nine plant families are susceptible to TMV virus [158].

VIGS is advantageous over other genome editing tools as it is a rapid and efficient method to study the gene function. As it is a transient method, there is no need to generate transgenic plants, screen large populations, and no plant transformations are required. As it is performed at the early stage of the plant, the target gene’s role in plant development is rapidly known. If a conserved sequence of the multigene family is chosen for the VIGS, it will silence all the genes in the family, and their role in the plant growth and development would be obvious. Otherwise, a gene member also can be targeted by choosing the sequence for VIGS. If the gene homologies are nearly the same, the same VIGS vector construct can be used to study the gene function in different plant species [56,156,158,159]. Most commonly studied genes using VIGS technology belong to defense responses and other developmental traits in different crop plants [160]. Several examples of RNAi in biotic and abiotic stress responses in tomato are presented in Tables 1–3.

### 2.4. Transposons

Transposable elements (TEs) or transposons are also known as “jumping genes,” are found in large proportions in most of the species’ genome. TEs were first reported as controlling elements in maize by Barbara McClintock in the 1950s [161]. TEs are one of the sources of the spontaneous mutations which can induce the genetic rearrangements in the genome, such as transposition, translocations, inversions, and duplication by excising from one place to another and integration into another chromosome [162,163] The TEs abundances vary from species to species. For example, TEs contribute to 80% of the maize genome, 66.8% of wheat, 38.8% of cabbage, 59% of soybean, 80% of barley, and 20% of *Arabidopsis thaliana* genome [164,165].

The transposons are classified into retrotransposons and DNA transposons based on the DNA and RNA intermediate. The retrotransposons are the Class I transposons that are amplified throughout the genome where RNA is reverse transcribed to cDNA and transpose themselves to different locations in the genome. The DNA transposons are the class II transposons where DNA is mobilized and integrated into other sites following a “cut and paste” mechanism in the genome via DNA intermediate [166,167]. The *Ac/Ds* (Activator/Dissociate) elements are class II transposons and were first identified in maize by Barbara McClintock in 1948, for which she was awarded a Nobel prize in 1983 [161,168,169]. Both the class I and II transposons have autonomous and non-autonomous elements. The autonomous elements use their own encoded proteins to mobilize transposons, while the non-autonomous uses the host machinery [167].

The plants generated through insertional mutagenesis using transposon DNA (T-DNA) are generally knockout mutants. It mostly creates a loss of function mutants, which will enable us to identify the gene function. Once the insertional mutants are generated, the transposed DNA will remain in the same location, even in the next generations. The T-DNA insertion may act as a marker for the identification of the mutant. T-DNA insertions have several disadvantages. If the T-DNA is placed in the intron, the insertion would be curated in the RNA splicing event. Furthermore, when the insertion of T-DNA is in the exon/intron splice site, it may lead to a truncated version of the protein. It may also lead to chromosomal dislocations [166].

The regulation of TEs in plant development is not well understood. However, few reports suggest the role of TEs in biotic and abiotic stresses. For example, *ONSEN Ty1-Copia* retrotransposon regulates the temperature stress while *Ty3-gypsy* retrotransposon mediates the escaping of epigenetic silencing [170,171]. In the genome of many plant species, TEs constitute a significant proportion. TEs would be better tools for improved crop breeding [172]. Insertional mutagenesis of T-DNA through *Agrobacterium*-mediated gene generates an inactivation gene, which is confirmed by a PCR-based approach called as site-selected insertion [173]. T-DNA insertions of maize transposable elements, activator/dissociation (Ac/Ds) have been exploited for insertional mutagenesis in tomato [55,174,175,176,177,178]. A study used site-selected insertion of *Polygalacturonase* (PG) and *Dihydroflavonol* 4-reductase (DHFR) in tomato [179]. This study showed that the progeny from *Ds* plants exhibited a high rate of insertion in PG than other genes [179]. Meissner and group [8] also created a similar mutant resource by using *Ac/Ds* elements in Micro-Tom and reported several lines carrying at least 2 or 3 *Ds* inserts. Similarly, several mutants sensitive to drought and salt stress have been identified in *Solanum pennellii* using T-DNA insertions [180]. Transposon tagging in tomato led to *FEEBLY* (*fb*) mutant isolation that displays high sensitivity to a herbicide called phosphinothricin [181].

### 2.5. Zinc Finger Nucleases (ZFNs)

**Figure 4:**
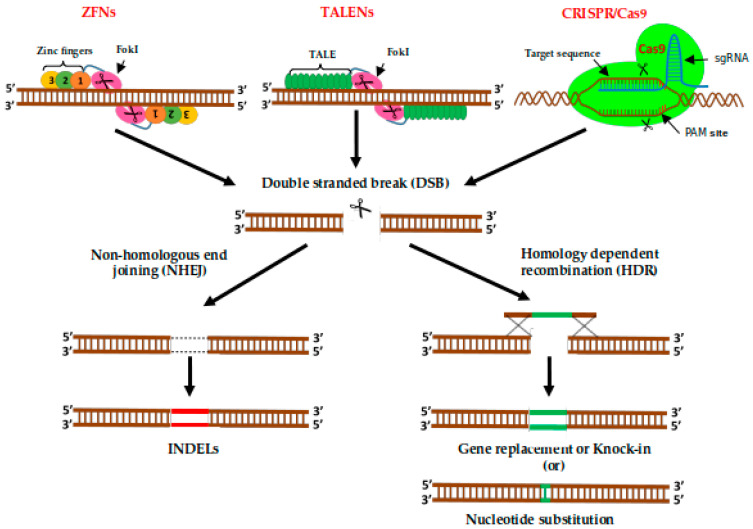
Represents the genome editing process in Zinc finger nucleases (ZFNs), transcription activator-like effector nucleases (TALENs) and CRISPR/Cas9. A pair of ZFNs bound to DNA. ZFNs are synthetic proteins with separate DNA-cleavage and DNA-binding domains, connected by a short linker sequence. By designing the recognition domain, it is possible to control the site of cleavage. The DNA-binding domain consists commonly of three ZFs. Each connects with 3 bp of target DNA. TALENs consist of a programmable DNA-binding domain and an unspecific *Fok*I cleavage domain. The TALENs recognition domain consists of two TALE DNA-binding sites, which contain arrays of multiple 34-amino acid repeat sequences. A single DNA nucleotide can be recognized by the amino acids at the 12th and 13th position. Or the CRISPR/Cas9 system, the first 20 bp guide sequence can quickly design and fused to the scaffold (gRNA backbone) sequence immediately upstream of a protospacer adjacent motif (PAM). The designed gRNA makes it possible to precisely guide the Cas9 RNA-guided endonucleases (RGENs) to induce a double-stranded DNA break (DSB) in the gene of interest. DBS in the target DNA is repaired either by NHEJ method resulting in small insertions or deletions (INDELs), or by HDR leading to knock-in or nucleotide substitutions.

Among the modern genome-editing tools, ZFNs are the pioneer that enable site-specific modifications in different organisms like the fruit fly, *zebrafish,* and plants such as *A. thaliana*, *N. benthamiana,* and *Zea mays* [182,183,184]. ZFNs have approximately 30 amino acids in length, which is stabilized by coordinating zinc ions to conserve the Cys2His2 motif [185]. The ZFN arrays can bind to the target DNA by inserting its α-helix into the DNA helix’s major groove and can recognize triple tandem nucleotides. Once ZFNs bind to the target DNAs, it introduces a double-stranded break in the DNA by its cleavage domain, *Fok*I restriction endonuclease [186]. The Fok I cleavage domain was isolated from the bacterium *Flavobacterium okeanokoites* [187] and must dimerize to catalyze DNA cleavage and become active. Therefore it is necessary to design two ZFNs, one for the complementary and one for the non-complementary DNA strand [188,189]. The DSBs activate the DNA repair system, which results in small insertions, deletions, or base substitutions [190] (Figure 4). After its discovery in 1996 [191], it has been successfully used in several plant species such as maize [184], tobacco [192], Arabidopsis [183,193] and soybean [194]. In *Z. mays*, exon 2 of *Inositol-1,3,4,5,6-pentakisphosphate 2-kinase* (*IPK1*) gene is disrupted, leading to herbicide resistance [184]. However, there is no literature available on tomato. 

Although, ZFNs are more specific resulting in fewer off-targets compared to CRISPR/Cas9 due to dimerization of *Fok*I domains [195], this technique also possesses several disadvantages. Unlike TALENs and CRISPR-Cas9, constructing zinc-finger arrays is difficult, hindering their widespread use in unspecialized laboratories [196]. Designing ZFNs is quite a cumbersome strategy and usually takes several months. As it is highly specific, a new cloning strategy is employed every-time, and is highly expensive [195,197,198].

### 2.6. Transcription Activator-Like Effector Nucleases (TALENs)

TALENs have emerged as an alternative tool for genome editing, similar to ZNFs [199]. Like ZFNs, TALENs also use *Fok*I domain as the DNA cleavage domain, cutting within a 12- to 19-bp spacer sequence that separates each TALE binding site; whereas the DNA binding domain consists of a tandem repeat of 33–35 amino acids, with highly variable amino acids at 12th and 13th positions [200,201] (Figure 4). These variable amino acids are referred to as repeat variable diresidue (RVD), which are specific to recognize particular nucleotides. The tandem repeat proteins from TALEs are effectors of the Xanthomonas bacteria, used to recognize DNA [200,201]. Unlike ZFNs, TALENs are easy to target any DNA sequence because of its simple interaction of the TALENS and DNA interaction [197,202]. However, there is a chance of off-targets in TALENS, which may lead to double-stranded break elsewhere in the genome [203,204]. In addition, TALENs are markedly larger than ZFNs, making their efficient delivery into cells challenging.

Nevertheless, TALENS is an efficient strategy to generate plants with efficient and economically improved traits [205]. TALENS has been successfully used in Arabidopsis [206,207], tobacco [208,209], rice [210,211], barley [212] and Brachypodium [213]. For example, in Arabidopsis, five genes (*ADH1, MAPKKK1, DSK2B, TT4,* and *NATA2*) were targeted using seven TALENS. They observed somatic mutations in the transgenic plants with a mutation frequency of 2–15% and the mutations were transferred to the transgenic progeny with a frequency of 1.5–12% [207]. The somatic mutagenesis was also reported in rice and barley [210,212]. High mutation frequency was reported in rice and generated transgenic plants that are resistant to plant pathogens [210]. In tomato, TALENS was used to edit *Anthocyanin gene* (*ANT1*), which encodes a MYB transcription factor [57]. Using Geminivirus for genetic transformation, they could obtain precise insertions with no off-targets at all. They also used CRISPR-Cas along with TALENs, which showed similar efficiencies in editing of *ANT1* gene. They overexpressed the *ANT1* transgene resulting in purple plant tissue [57].

### 2.7. CRISPR/Cas9

CRISPR/Cas9 is a rapidly emerging genome editing tool, immensely used in various organisms, including plants. Unlike ZFNs and TALENs, CRISPR/Cas9 made the genome editing much convenient and effective to generate knockout mutants [190]. ZFNs and TALENS use *Fok*I endonuclease, which forms a dimer for better specificity to bind to the target DNA. The designing of active *Fok*I nucleases is tedious and very expensive. CRISPR/Cas9 requires a guide RNA (gRNA) to target the gene and a Cas9 endonuclease, which is an RNA dependent DNA endonuclease to edit. Cas9 forms a complex with gRNA and recognizes a specific protospacer adjacent motif (PAM) with a consensus sequence of 5′ NGG 3′ at the 3′ end of the target sequence. The Cas9 induces a double-stranded break, 2–5 bp upstream of the PAM [211,214,215].

The basic principle of CRISPR/Cas9 is taken from a bacterial immune system where DNA segments of the invaded virus are arranged in an array called CRISPR array [216,217]. Upon exposure to the virus again, the bacteria generate RNA segments of the CRISPR array to bind and destroy the genome of the virus [214,218,219,220]. The Cas9 protein is composed of two connected lobes, a nuclease (NUC) lobe, and a recognition (REC) lobe. The NUC lobe has a PAM-interacting (PI) domain and two nickase domains, HNH is responsible for cleavage of the complementary strand and RuvC responsible for cleavage the non-complementary strand. The REC lobe is responsible for Cas9-sgRNA complex formation. During gRNA loading the confirmation of Cas9 changes from an inactive to an active form by building a central channel where the RNA-DNA heteroduplex will be positioned. The Cas9-gRNA complex scans the double-stranded DNA. Once the PI domain recognizes a three-bp-long PAM sequence, the DNA is melted, and the complementary DNA strand connects with the gRNA (RNA-DNA heteroduplex formation). After heteroduplex formation, the HNH and RuvC nickase domains cleave the double-stranded DNA three bases upstream of the PAM sequence [221,222,223] (Figure 4).

According to [224], the CRISPR/Cas systems are separated into two classes with several types and subtypes. The class 1 CRISPR systems (type I, III, and IV) use numerous Cas proteins to form a complex with crRNAs (tracrRNA), whereas the class 2 CRISPR systems (type II, V, and VI) use a single Cas protein. The Cas9 enzyme commonly used in most CRISPR studies, belongs to type II, is acquired from *Streptococcus pyogenes* [214]. The CRISPR/Cas induces a DSB at the target gene, which is repaired by the DNA repair system of the host [214,225,226]. The CRISPR knockouts generated are looked for the presence of the mutation and the removal of the transgene. Although CRISPR/Cas9 is very precise, it is not an unfailing system. Unexpected mutations, so-called off-targets effects, could be detected in various studies, which lead to concerns for biomedical and clinical applications [227]. However, the off-target effects in plants are very rare [228]. Nevertheless, different strategies were developed to increase the specificity of Cas9 cleavage (e.g., using double nicking, or truncate gRNA).

Compared to ZFNs and TALENs, the CRISPR/Cas9 editing system is easy to perform as it requires gRNA of 18–20 bp while ZFNs and TALENs need designing of specific nucleases, which is cumbersome and laborious. Besides gene knockout, the CRISPR/Cas9 tool can be used for several applications. The deletion of whole gene clusters is possible by the simultaneous expression of two or more gRNAs. Due to the cell’s homology-directed repair (HDR) of DSBs, genes can be knocked-in by providing template DNA with overlapping flanking regions [221,229]. For CRISPR/Cas9 system, the major limitation is the PAM motif requirement. The sporadic presence of PAM sequence in the genome or sometimes its complete absence in the desired coding parts of the genomes to be edited restricts the application of CRISPR/Cas9 [214,230]. To be more versatile by searching target sites in the gene of interest it is possible to use Cas9 proteins from other bacteria like *S. aureus*, *S. thermophilus, Neisseria meningitides,* or *Brevibacillus laterosporus*, which have different PAM specificity [231,232,233,234]. Another CRISPR tool specific to the RNA is CRISPR/C2c2, a type VI CRISPR/Cas system [235,236].

Being precise, efficient, and highly cost-effective technology, CRISPR/Cas9 is extensively used to edit several crop plants since its first report in 2013. Since then, this technology has been revolutionizing crop breeding. Several studies have reported genome editing using CRISPR/Cas9 in Arabidopsis, tobacco, rice, cucumber, maize, tomato, wheat, cassava, and potato to develop improved crop varieties in terms of biotic and abiotic stress responses as well as nutritional and other aspects [237]. Some of the most prominent examples of CRISPR/Cas9 editing are listed in Tables 1, 2 and 4.

## 3. Genome Editing for Resistance Breeding in Tomato

### 3.1. Biotic Stress Resistance Breeding in Tomato

The biotic factors include diseases caused by the attack of pathogens that reduces the crop yield by 20–40% worldwide (http://www.fao.org/news/story/en/item/280489/icode/). Several strategies have been developed for controlling diseases. The pesticides are used to contain the pathogens, but the pesticide spray is harmful to both humans and the environment [238]. The spray of chemicals also destroys some useful organisms, hence disturbing the ecological balance. In order to overcome diseases, the development and use of more pesticides are often exploited as a preventive measure. However, the application of pesticides is successful in resisting pathogens to some extent. Therefore, the dependence of crop productivity on chemicals/pesticides is not the best solution due to their harmful side-effects that may lead to ecological imbalance over long-term usage [239].

Genetic breeding of disease resistance crops provides an efficient and eco-friendly strategy to resist diseases. Initially, conventional breeding methods were successful in creating genetic resources with disease resistance. However, there are various disadvantages to traditional breeding. Firstly, it can be used for plants that can mate with each other. Secondly, the plant population must have sufficient genetic variation, especially in disease resistance. Thirdly, the time required and linkage drag often introduces several unwanted traits such as decreased yield. It is challenging to keep pace with fast-evolving pathogens with conventional breeding techniques [240,241].

The plants produced by targeted gene editing can be exploited to overcome the above challenges. The genes that negatively regulate disease resistance represent promising targets for genome editing. The examples of biotic stress responses in tomato are listed in Table 1.

#### 3.1.1. Fungal Infections

Some of the most common and prevalent fungal infections in tomato are gray mold (*Botrytis cinerea*), early blight (*Alternaria solani*), late blight (*Phytophthora infestans*), and Fusarium wilt (*Fusarium oxysporum* f. sp. *lycopersici*). Here are a few examples of disease resistance for fungal infections using genome editing (Table 1).

*B. cinerea* is the most cosmopolitan and destructive fungal infection that causes gray mold in several plants, including tomato. Buxdorf et al. [242] have reported that RNAi silencing of *SlSHN3* TF resulted in reduced susceptibility to the necrotrophic foliar pathogen *B. cinerea* in Micro-Tom tomato. In contrast, over-expression of *SlSHN3* caused resistance to *B. cinerea* infection. Another similar study reported reduced tolerance to *B. cinerea* upon RNAi silencing of tomato *pectate lyase* (*SlPL*) [243]. *SlNL33* is a gene that encode an NL type of NBS-LRR resistance proteins. Silencing of *SlNL33* not only increased the tolerance to gray mold disease by *B. cinerea* but also enhanced the tolerance to oxidative stress [244].

The fungus *F. oxysporium* is a soil-borne parasite that penetrates the vascular tissues of the roots through hyphal branching and invades the xylem causing a characteristic wilting phenotype. This fungus destroys many economically important crops. *Fusarium mitogen*-activated protein kinase (MAPK) signaling genes (*FMK1, HOG1*), and *PBS2*) are involved in plant surface hydrophobicity (sensing) and pathogenesis [245]. The silencing of these three genes in *F. oxysporium* resulted in reduced mycelial growth on tomato fruits leading to reduced pathogenicity compared to the unsilenced fungus [246]. Zhang et al. [247] reported that knockout mutants of *SlMAPK3* are susceptible to grey mold disease.

Prihatna et al. [248] reported the involvement of a novel gene expressed in the roots of reduced mycorrhizal colonization (*rmc*) mutant. This mutant is incapable of forming mycorrhiza colonization but is vulnerable to Fusarium wilt. The *rmc* mutant has a chromosomal deletion of five genes, where one of them is CYCLOPS, which is non-mycorrhizal similar to *rmc* mutant but is not susceptible to *Tfw*. Solyc08g075770 is one of the other four genes in the deleted fragment. This gene function is not yet annotated but is expressed in roots. CRISPR/Cas9 knockouts of this gene resulted in some mutants with *rmc* phenotype (susceptible to *Tfw*) and *rmc* mutants with 76R (wild type) phenotype.

Nekrasov et al. [249] generated a CRISPR/Cas9 mutant of Mildew resistance locus (*Mlo*) in Moneymaker. The wild type allele of *Mlo* is susceptible to powdery mildew, whereas the knockout mutants of *mlo* are resistant to the fungus causing powdery mildew infection. In a similar study, CRISPR/Cas9 mutants of Powdery Mildew Resistance 4 (*PMR4*) were resistant to the powdery mildew infection [250]. RNA silencing of *SlPMR4* also showed enhanced resistance to powdery mildew [251]. A recent study used four guide RNAs to generate a full knockout CRISPR/Cas9 mutant of *SlPMR4*. However, the knockout mutants exhibited reduced susceptibility to the fungus *Oidium neolycopersici* but not complete resistance.

#### 3.1.2. Viral Diseases

Several viruses like Gemini viruses are a threat to most of the crop plants, which affect crop productivity. Here are a few examples. Tashkandi et al. [252] targeted the coat protein (CP) and replicase (Rep) of *Tomato Yellow leaf Curl Virus* (TYLCV) using the CRISPR/Cas9 system in tomato cultivar Moneymaker. The CRISPR edited plants were resistant to the TYLCV infection, which was apparent by low levels of the TYLCV genome in the edited plants. The CP region of the genome was efficiently targeted than Rep. It could be due to the competition between the viral replication machinery and the Cas9 endonuclease to the Rep.

Another viral pathogen causing significant damage to tomato and other crop plants is *Tomato leaf curl virus* (ToLCV), which is transmitted by whitefly. In a study by [253], intron-hairpin RNA was used for silencing the ToLCV infection. Seven hundred and twenty-seven nucleotides at the *C1* gene were used in sense and antisense orientation separated by an intron to form a hairpin. *C1* gene is the ToLCV replication-associated protein. RNAi silencing of the *C1* gene resulted in resistance against ToLCV. Similarly, siRNA mediated silencing of conserved sequences of ToLCV antisense replicase (*AC4*) gene efficiently inhibited the geminivirus ToLCV in the most ToLCV sensitive tomato cultivar, Campbell [254]. A related study of the silencing of *Rep* gene of ToLCV showed that a threshold level of dsRNA is needed for gene silencing resulting in inhibition of the virus in the ToLCV-infected tomato plants [255].

#### 3.1.3. Bacterial Diseases

*Pseudomonas syringae* is a cosmopolitan bacterial pathogen affecting the most important crop plants. Upon infection, *P. syringae* pv. Tomato (*Pto*) DC3000 releases coronatine (COR) that induces a stomatal opening for the bacteria invasion. COR needs a coreceptor JAZ2 for a stomatal response. CRISPR/Cas9 edited *SlJAZ2* lacked Jas domain at the C-terminal end of JAZ2, which is now *SlJaz2* Δjas, which are resistant to the bacterial speck disease [256]. The study also conferred resistance to necrotrophs such as *B. cinerea* by uncoupling SA-JA pathways [255].

#### 3.1.4. Oxidative Stress

*Ralstonia solanacearum* is a soil-borne pathogen that infects the host roots to cause a bacterial wilt disease. The pathogen infests through mechanical injury or wounds. Once infected, the pathogen enters the xylem of host roots to acquire the essentials nutrients leading the plant to death. The host roots release reactive oxygen species (ROS) in response to stress. As a result, the pathogen *R. solanacearum* experiences oxidative stress. Then pathogen upregulates the *DPS* gene (DNA binding protein from starved cells) to acclimatize to the host’s conditions. The DPS helps to maintain DNA integrity under stressful situations, including lack of nutrients, in *E. coli* and also protects the bacteria from oxidative stress [257,258,259,260,261,262,263]. The *dps* mutant (insertional) exhibited increased hydrogen peroxide levels and increased mutation rate upon starvation. The oxidative stress response regulator (OxyR) upregulated the expression of dps. *R. solanacearum* requires DPS to overcome the oxidative stress in the host roots, and the mutant shows decreased virulence in the host [264].

A tomato HD-ZIP1 TF (SlHZ24), regulates the transcription of GDP-D-mannose pyrophosphorylase 3 (*SlGMP3*) by binding to the promoter element of *SlGMP3*. The over-expression of *SlGMP3* increased the ascorbic acid A levels by 1.6-fold. The ascorbic acid is produced under stress conditions to protect the plants from ROS. The over-expression of *SlHZ24* elevated the ascorbic acid in the transgenic plants improving stress tolerance. While RNAi silenced, plants are sensitive to oxidative stress [265].

### 3.2. Abiotic Stress Resistance Breeding in Tomato

The abiotic stress conditions include drought, salinity, light intensity, temperature (hot/cold), nutrient availability, and biotic stress that involve an attack by pathogens. The examples of abiotic stress response are listed in Table 2.

#### 3.2.1. Freezing Stress

Ethylene response factors (ERF) family members play a role in plant stress responses [276], Briefings in functional biology). In tomato, TERF2/LeERF2 regulates the ethylene biosynthesis transcriptionally and is induced by cold temperature [277,278]. In tobacco, the over-expression of *TERF2/LeERF2* enhanced the tolerance to freezing with increased expression of cold response genes. While RNAi silenced lines showed decreased expression of cold-responsive genes and reduced tolerance to freezing stress [279].

#### 3.2.2. Chilling Stress

Cold temperatures affect plant growth and development, limiting crop productivity. The invertases convert sucrose to glucose and fructose. These hexoses act as signals molecules in plant stress responses [280,281,282,283]. Tomato *cell wall invertase inhibitor* (*SlINVINH1*) inhibits the invertase activity, thus limiting hexose production. Cold stress suppressed the expression of *SlINVINH1* and induced the expression of invertases (Lin6 and Lin8). The RNAi mediated silencing of *SlINVINH1* increased the invertase activity and improved the tolerance to chilling, whereas over-expression of *SlINVINH1* suppressed the cell wall invertase activity, making the plants sensitive to cold stress. Glucose is known to induce the expression of ABA biosynthesis and signaling genes. Therefore, they checked for the expression of tomato *9-cis-epoxy carotenoid dioxygenases1* (*SlNCED1*) in silenced *SlINVINH1* mutants and observed that cold stress-induced the *SlNCED1* expression in the silenced lines and wild type [284].

Fructose 1,6 biphosphate aldolases (FBA) play an important role in the Calvin-Benson cycle and FBA expression vary with response to heat or cold stress in tomato seedlings [285,286,287]. Eight FBA genes are present in tomato. All FBA members are susceptible to cold temperatures in tomato seedlings [285]. Cai et al. [288] suppressed the *SlFBA7* and observed that the RNAi silenced lines were found to show severe injury symptoms in response to chilling stress, which could be due to increased levels of hydrogen peroxide (H_2_O_2_) and superoxide anions (O_2_-). Similar studies of silenced plants of several other genes show susceptibility to chilling stress (Table 2), which could be possible candidates to overcome the chilling stress.

#### 3.2.3. Heat Stress

Like cold, heat stress is also major environmental stress to plants affecting plant growth and development. In the coming years, heat stress is a serious threat to the environment due to global warming, making climate warmer [289]. Plants evolve mechanisms to tolerate these adverse conditions. In response to these situations, several genes responsive to heat stress have been identified and are summarized in Table 2.

As discussed in the above section (biotic stress), MAP kinases are involved in plant growth and developmental processes, disease resistance, and various stress responses such as drought, salt, and cold. The first report describing the role of MAPKs in heat stress came from the study of Link et al. [290]. Heat stress by activating MAP kinase was found to regulate tomato plant response by phosphorylating heat stress TF *HsfA3*. Similarly, in Arabidopsis, MAPK activates the *HsfA2* in response to heat stress [291]. In a recent study, RNAi silencing of *SlMPK1* (*MAP kinase1*) in tomato resulted in improved heat tolerance [292]. In contrast, its over-expression resulted in a compromised tolerance. Likewise, Mishra et al. [293] used the post-transcriptional gene silencing of *SlHsfA1* by short interfering RNAs. Tandem inverted repeats of *SlHsfA1* was used for co-suppression. The co-suppressed lines of *SlHsfA1* exhibited sensitivity to heat stress, while over-expressor lines showed enhanced tolerance to heat.

The plant hormone brassinosteroids (BRs) play an important role in various plant developmental and physiological processes such as seed germination, photomorphogenesis, cell elongation, cell division, xylem differentiation, and plant reproduction [294]. BRs also play a role in biotic and abiotic stress responses [295,296,297]. BR signaling is majorly regulated through two TFs, Brassinazole resistant1 (BZR1) and BZR1-EMS-suppressor1 (BES1 or BZR2). CRISPR/Cas9 knockout plants of *SlBZR1* was susceptible to heat stress with a decreased quantum efficiency of PSII. In response to heat stress, ROS and heat shock proteins are induced but downregulated in the knockout mutant. It also led to the curtailed production of apoplastic H_2_O_2_ (which activates the ROS-scavenging enzymes) in the knockout mutant in response to heat stress [298].

#### 3.2.4. Drought Stress

ABA is conjugated to glucose by uridine diphosphate glycosyltransferases (UGTs) and is required for ABA homeostasis [299]. Among three UGTs in tomato, [300] silenced *SlUGT75C1* using RNAi and observed that *SlNCED1* (key enzyme in ABA biosynthesis) levels were unaltered while *SlCYP707A2* (a key enzyme in ABA degradation) was up-regulated in the silenced line. Consistent with the down-regulation of *SlUGT75C1* in the silenced line, ABA levels significantly increased, whereas Glc-conjugated ABA (ABA-GE) levels were decreased. Increased ABA and ethylene in the silenced fruits hastened fruit ripening. The knockdown mutants also exhibited tolerance to drought stress.

bZIP has an important role in various physiological and signaling processes and also in stress response. bZIP proteins such as ABA-responsive element-binding proteins (AREBs) and ABRE binding factors (ABFs) regulate the ABA-dependent transcription and abiotic stress responses [301,302]. ABA and salt stress induce the expression of *bZIP1*. RNAi silencing of *bZIP1* downregulated the genes involved in ABA biosynthesis such as *SlNCED1*, *SlNCED2*, and signal transduction genes such as *SlABF2*, *SlABF4* were down-regulated. Further, the silenced plants exhibited reduced tolerance to both drought and salt stresses [303]. Therefore, as the bZIP act as essential regulator of stress tolerance, these could be promising candidates for crop improvement in response to stress.

#### 3.2.5. Salt Stress

A tomato MADS-box TF, *SlMBP11*, is implicated in tolerance to salinity [304]. Similarly, *SlMBP8* is involved in abiotic stress responses such as salinity, drought, wounding, cold, and heat stresses. *SlMBP8* is also induced by methyl, jasmonates, 1-amino cyclopropane-1-carboxylic acid, indole acetic acid, and ABA [305]. RNAi knockdown lines of *SlMBP8* showed improved tolerance to drought and salinity [305].

SlZF3 is a C_2_H_2_ zinc-finger protein TF. Zinc finger TFs are involved in plant stress response/tolerance. Salt stress induces the expression of *SlZF3*. RNAi silenced lines of *SlZF3* are susceptible to salt stress, whereas its over-expression lines are tolerant to salt stress [306].

To elucidate the role of GABA in salt stress, Bao et al. [307] silenced the GABA pathway genes, Glutamate decarboxylase (GAD), GABA transaminase (GABA-T), and succinic semialdehyde dehydrogenase (SSADH) using the VIGS approach. Silencing of *SlGAD* and *SlGABA-T* showed increased sensitivity to salinity, while that of *SlSSADH* showed reduced sensitivity to salt stress.

#### 3.2.6. Osmotic Stress

Osmotic stress is mainly caused by drought, salinity, and cold stresses where the plant cells lose its water potential, reducing crop productivity [308]. The osmotic stress involves oxidative damage and ROS production. Excessive production of ROS induces cell damage and tissue death [309]. The osmotic stress causes the SNF1-related protein kinase2 (SnRK2) family, which are essential for stress signaling. SnRK2 family has seven genes out of which *SnRK2.1*, *2.2*, and *2.3* were suppressed while the expression of the remaining four members of the family was initially decreased then increased [310]. The over-expression of *SnRK2.1* and *SnRK2.2* resulted in plants with reduced tolerance to salt stress, while RNAi silenced lines showed enhanced tolerance to salt stress [310].

Another study by Borsani et al. [311] screened EMS population based on sensitivity to osmotic stress. They isolated two osmotic hypersensitive mutants, *tomato osmotic sensitive-1* (*tos1*) and *tomato salt sensitive-2* (*tss2*) [311]. When checked for seed germination, tos1 showed reduced sen-sitivity to ABA while *tss2* was hypersensitive to ABA. The study further suggests that ABA perception and signaling are essential for osmotic stress response [311].

### 3.3. Genes Regulating Combined Biotic and Abiotic Stress Response

There are very few studies on tomato where editing of genes regulated both biotic and abiotic stresses. Here we discussed the selected examples below and further summarized the entire list in Table 3.

NAC TFs are involved in various plant growth and developmental processes and participate in response to various biotic and abiotic stresses. Liu et al. [337] identified *SlSRN1* (Stress Related NAC1). Infection of tomato plants with *B. cinerea* and *Pto* DC3000 triggered *SlSRN1* expression. Silencing of *SlSRN1* through VIGS resulted in more susceptibility of plant infections caused by *B. cinerea* and *Pto* DC3000. However, the VIGS silenced plants were more tolerant to drought and oxidative stress [337].

Another gene, *SlTomLoxD*, is a lipoxygenase that plays a key role in jasmonic acid (JA) biosynthesis [338]. The expression of *SlTomLoxD* is induced in response to wounds and pathogen infections by stimulating JA and systemins [339]. RNAi silencing of *SlTomLoxD* resulted in reduced susceptibility to heat and highly susceptible to *Cladosporium fulvum* [340].

AbuQamar et al. [341] reported that ABA-induced R2R3MYB1 (AIM1) TF is induced in response to oxidative stress, pathogens, salt stress, and plant hormones. RNAi silenced plants of *SlAIM1* were highly sensitive to salinity and oxidative stress and also susceptible to *B. cinerea*.

A family of transcription activators, called SR/CAMTA (signal responsive/Calmodulin transcription activators), are involved in the response to biotic and abiotic factors. In Arabidopsis and rice, these proteins play an important role in response to several biotic factors such as *Golovinomyces cichoracearum*, *P. syringae* pv. tomato (*Pto*) DC3000 and *B. cinerea* [342,343,344,345,346] and abiotic factors such as temperature and drought [347,348,349,350]. In tomato, seven members constitute this gene family [351]. VIGS silencing of *SlSR1L* led to increased susceptibility to drought stress along with the reduced expression of drought-responsive genes [352]. Similarly, VIGS mediated silencing of *SlSR1*, and *SlSR3L* genes exhibited enhanced tolerance to necrotrophic fungus *B. cinerea* and *Pst* DC3000 [352].

Hybrid proline-rich proteins (HyPRP) are well-known proteins in plant response to various biotic and abiotic factors [353,354,355,356,357]. In tomato, HyPRPs are downregulated under stress conditions such as heat, cold, salt, drought, oxidative stress, etc. Li et al. [358] reported that RNAi knockdown plants showed increased resistance to salt, dehydration, and oxidative stress.

*SlPti4* is a tomato TF of the ERF gene family, and it plays an essential role in plant disease resistance [359,360,361]. The RNAi knockdown of *SlPti4* resulted in the transgenic plants with decreased tolerance to drought and enhanced susceptibility to *B. cinerea* [362].

### 3.4. Genome Editing for Yield and Fruit Quality Improvement in Tomato

Fruit quality and quantity are certain key traits that are necessary for crop improvement. While early flowering, multiple flowers, determinate growth habit, fruit size, and locule number are the major traits deciding the yield, fruit quality and nutritional value are rated based on fruit ripening, fruit pigmentation, and fruit metabolites. The examples of fruit quality and yield improvement in tomato using genome editing are listed in Table 4.

Many genome editing experiments resulted in a change in floral morphology and flowering time. Xu et al. [363] identified one EMS mutant for *fab* (*fasciated and branched*) and two EMS mutants, and three fast neutrons (FN) mutants for *fin* (*fasciated inflorescence*). These mutants were characterized with enlarged meristems resulting in branched inflorescence and fasciated flowers, leading to fruits with a higher number of locules. Map-based cloning identified the mutations in *CLAVATA1* (*CLV1*) for *FAB* and an *arabinosyltransferase* gene for *FIN*. To generate quantitative variants by editing the Cis-regulatory elements (CRE), Rodríguez-Leal et al. [364] generated CRISPR/Cas9 mutants in *S. pimpinellifolium*. Mutations in the promoter of *CLV3* resulted in a series of variants for floral organs and locule number. They also generated promoter mutants for *WUSCHEL-RELATED HOMEOBOX 9, WOX9* (*S*), and *SELF PRUNING* (*SP*), resulting in many quantitative variations in plant architecture and inflorescence modifications. These variants allow studying specific CREs in the regulation of gene expression. They are also promising sources to explore the dominant and semi-dominant effects of genes with possibilities for orphan crops’ domestications. CRISPR/Cas9 mediated gene editing for the flowering repressor *SELF-PRUNING 5G* (*SP5G*) leads to compact determinate growth habit, with day-length-independent early flowering facilitating early harvest [365]. They introduced the mutation into the *sp* background, which has a determinate growth habit, resulting in ‘double-determinate’ plants with early yield. This study was later supported by another similar gene-editing effort by [366]. Since *S. pimpinellilfolium* is tolerant of many bacterial diseases and salt stress, they generated single and double mutants for *SP* and *SP5G* through CRISPR/Cas9-engineering, confirming the effect of *sp* in determinate growth habit, *sp5G* in day neutrality, and the double mutant with synchronized fruit ripening. They also generated mutants targeting the promoter region of *WUSCHEL (WUS)* and *CLV3.* While mutation in the *WUS* promoter enlarged the fruit size, the CLV3 promoter mutation did not increase the locule numbers as expected, possibly because the targeted site was not necessary for gene expression regulation. This study’s highlight was that the retention of the disease and salt tolerance of *S. pimpinellilfolium* in the CRISPR/Cas9 mutants. Efforts to domesticate *S. pimpinellilfolium* was also carried out by Zsögön et al. [367] using multiplex CRISPR/Cas9 editing of coding sequences of *SP*, *OVATE* (*O*), *CLV3*, *MULTIFLORA* (*MULT*), and *LYCOPENE BETA CYCLASE* (*CycB*). This multiplex editing resulted in diverse combinations of mutant alleles. The possibility of off-target editing was excluded by sequencing of two closely related targets for each gene. This study successfully introduced several domestication traits, including determining growth habits, more fruits (10-fold increase) with a bigger size (3-fold) of the fruits, and a 500% increase in fruit lycopene content [362]. 

Fruit ripening is another important fruit trait that has been targeted using CRISPR/Cas9 mediated gene editing. Ito et al. [368] targeted three regions in the coding sequence of the MADS-box TF *RIN* (*RIPENING INHIBITOR*), resulting in the generation of multiple mutated alleles with altered ripening phenotypes ranging from low pigmentation and incomplete ripening in varying degrees. The actual *rin* mutant was thought to be a null mutant, but the CRISPR knockout of RIN is rather a gain of function mutant repressing the fruit ripening process [369]. The knockout mutant of RIN shows induction of fruit ripening associated with physiological changes such as lycopene accumulation albeit much lower than wild type and expression of fruit softening enzymes such as pectate lyase and polygalacturonase. This study suggests that RIN is essential for fruit ripening but not for induction of fruit ripening [370]. Virus-induced gene silencing of 11 putative tomato fruit ripening-related factors indicated that *organelle RNA recognition motif-containing protein4* (*ORRM4*) positively regulates tomato fruit ripening. The coding sequence of *SlORRM4* was edited using the CRISPR/Cas9 method, leading to a delay in fruit ripening associated with low ethylene production and respiratory rate. This study concluded that *ORRM4* is a Mitochondria-localized RNA editing factor that has a significant role in regulating the fruit ripening process [371].

The long shelf life of the fruits is another desirable trait in tomato. Yu et al. [372] reported knock-out of *ALC* (*Alcobaca*) gene using CRISPR/Cas9 mediated editing, resulting in delayed pigment accumulation. However, it did not affect the fruit ripening, harvest time, or the ripened fruit’s color. Most importantly, it was observed that *alc* mutation significantly improved the fruit shelf life. They also generated Cas9-overexpressing (Cas9-OE) *S. lycopersicum* cv. M82 transgenic lines for “virus-mediated genome editing system”, where a TRV RNA2 genome-derived vector facilitates the gRNA delivery. Another study reported that *SlGH3.2* expression is induced during fruit ripening. The RNAi silencing of *SlGH3.2* elevated the shelf life of fruits possibly due to increased IAA and IBA levels in the transgenic fruits [373].

Klap et al. [329] identified a parthenocarpic mutant from an EMS mutagenized population of M82 cultivar. They identified a candidate gene *AGAMOUS-LIKE 6* (*SlAGL6*) and created gene knockout lines targeting the coding sequence using CRISPR/Cas9. The mutant exhibited facultative parthenocarpy, which resulted in fruit production under heat stress conditions. Another gene-editing study on *SlIAA9* resulted in parthenocarpy and altered leaf morphology [374].

There are few gene-editing studies carried out for carotenoids and anthocyanins in tomato. Hayut et al. reported the *PHYTOENE SYNTHASE (PSY1)* gene-editing resulting in yellow fruits with red sectors. They also demonstrated somatically induced double-strand breaks by crossing the *psy1* mutant with *S. pimpinellifolium*. Multiplex CRISPR/Cas9 genome editing was carried out for *stay-green 1* (*SGR1*), *lycopene ε-cyclase* (*LCY-E*), *beta-lycopene cyclase* (*Blc*), *lycopene b-cyclase 1* (*LCY-B1*), and *LCY-B2* in *Solanum lycopersicum* cv. AC [375]. This resulted in various combinations of mutations. The knock-out lines with only *SGR1* mutation exhibited the highest lycopene (5.1-fold) and increased other carotenoids like phytoene, prolycopene, α-carotene, and lutein compared to the remaining combinations. All the mutants with the *SGR1* mutation showed the characteristic rust color after the breaker stage. In another study, fast neutron-induced mutagenesis, followed by map-based cloning, identified a *tangerine* mutant with a 282 bp deletion in the coding sequence [14]. Čermák et al. [57] reported the use of both TALENs and CRISPR/Cas9 to edit *Anthocycanin1* (*ANT1*) gene, which codes for an MYB TF using geminivirus replicons. Instead of silencing, they overexpressed the endogenous *ANT1* coding sequence by inserting a cauliflower mosaic virus 35S promoter upstream. They could achieve precise insertion without sequence modifications in more than two-thirds of the insertions. Almost the same efficiency was observed for both the methods without any off-target modifications. Moreover, these chromosomal changes showed a Mendelian pattern of transmission to the next generation. Transposon mediated mutagenesis of *FEEBLY* (*FB*) gene with an insertion of the *Ds* element in the intron was characterized by high anthocyanin levels, small, fragile plants, and insensitivity to phosphinothricin [181].

ZFNs-mediated genome editing for *LEAFY-COTYLEDON1-LIKE4* (*L1L4*) resulted in a number of phenotypic variations, including seed storage proteins and fatty acids, fruit shape, moisture content, and fruit metabolite levels including fructose, total polyphenols, antioxidants, β-carotene, oxalic, and citric acid [376,377]. Some other CRISPR/Cas9 gene-editing studies resulted in enhanced GABA content and ascorbic acid content in leaves [378], changes in leaf complexity, shape, and serration [61,379,380]. Two independent reports explained the knockdown of *PDS*, resulting in the plant’s albino phenotype [366,381]. Transposon-mediated mutagenesis was achieved for *defective chloroplasts and leaves* (*DCL*) gene resulting in albino phenotype with green patches [382]. Ron et al. [383] reported the short root phenotype by editing the *SHR* gene. TALENs mediated knockout of the *PROCERA* gene resulted in enhanced GA response in tomato [384].

## 4. Challenges and Future Perspectives

Tomato has been domesticated by selective breeding that often leads to loss of genetic diversity and fitness. Conventional breeding of desired traits into the elite tomato cultivars is quiet a labor-intensive and time-consuming process. The availability of tomato genome sequence has increased the use of reverse genetic tools such as induced mutagenesis, transposons, RNA interference, etc. However, these tools have several limitations such as screening of large populations, cloning and transformation strategies, which are quite labor-intensive and cumbersome. These challenges were overwhelmed by genome editing tools such as ZFNs, TALENs, CRISPR/Cas9 in crop plants such as tomato. These tools offer precise editing of desired genes and multiple genes can be edited simultaneously, which can accelerate the breeding process and greatly reduce costs. However, the genome editing tools also possess disadvantages. For example, TALENs cannot edit methylated regions and the engineering of endonuclease every time is the major limitation in ZFNs and TALENs. CRISPR/Cas9 does not require engineering of Cas9 to target different genes. However, the PAM sequence which is usually 2–5 bp, is found very often in the genome, therefore it is difficult to edit the desired genes with the sequence constraint [386]. This can be overcome by variants of Cas9 with different sequence specificities for PAM. For instance, Cas12a (Cpf1), a class II Type V nuclease requires a PAM sequence rich in thymine (5′-TTTN-3′) [387,388]. The genomes such as tomato are AT-rich, and certain genomic regions are reluctant to gene editing. Such AT-rich regions can be edited by Cas variants like Cas12a. The generation of off-targets has also been a major concern in genome editing and the use of Cas12a is said to decrease the possibility of off-target mutations [53]. The induction of double cas9 nickase mutants or the truncation of the gRNA increase the specificity of Cas9 cleavage and reduce the off-target effects. Besides, to perform precise genome editing or to introduce new elements, it is essential to overcome the limitation of the low frequency of HDR in plant [227].

As a result of selective breeding, much of the tomato genetic diversity is lost in the modern domestic cultivars. This can be restored by introgression of specific traits into the domesticated cultivars, but since this process is quite laborious, genome editing can be useful to help restore the lost genetic traits in the present tomato cultivars. There are various examples of gene editing in tomato that are presented in this review, which show stress resistance and improvement in yield and nutrition. However, significant progress is required to achieve success in combating the changing environmental conditions. New variants of Cas9 with diverse specificities of PAM would greatly enhance the genetic restoration in crop plants such as tomato.

A combination of genome editing technology and conventional breeding can speed up the introduction of the trait of interest. Genetic crosses help remove undesired elements, which is a prerequisite to gain regulatory approval of transgene-free gene-edited plants [389]. Apart from these, there are several regulatory issues associated with genome-edited plants. There exists a gap in public towards the understanding of gene edited plants with and without foreign genetic material. There are several hurdles in releasing the non-GMO plants to the public, therefore the regulatory policies need to be harmonized. In 2016, the United States Department of Agriculture gave an exception to genome-edited mushrooms and corn from the traditional genetic modification policies, whereas the Court of justice of the European Union announced that the gene-edited crops come under genetically modified organisms. Recently, the United States Department of Agriculture released six virus-resistant tomato plants generated by gene-editing (https://www.isaaa.org/kc/cropbiotechupdate/article/default.asp?ID=17661).

## 5. Conclusions

According to the Food and Agricultural Organization, the world population would reach approximately 9 billion by 2050. It is estimated that crop productivity must be increased by 70% to feed the ever-increasing population (United Nations World Population Prospects: The 2017 Revision; FAO, 2019). New varieties of vegetables with high yield and stress-tolerance must be developed to address food scarcity for the overexploited population under an ever-changing climate [390]. Over time, humans have successfully developed crops with new or improved traits by transferring desirable genetic variations through conventional breeding techniques. Although conventional breeding methods have improved gradually, there is still an urgent need to enhance crop yield and productivity further quickly in the recent future [391,392].

With the advent of sequencing technologies, functional genomics has revolutionized with new editing tools to create novel allelic series of mutants for crop improvement. Among them, CRISPR-Cas technology has gained much attention over the other genome editing techniques because designing specific nuclease domains each time is a tedious task. However, in the last decade, CRISPR has emerged with different Cas endonucleases rendering the editing process much more precise and easier. In tomato, the genome editing tools is applied to enhance the nutritional value, yield, and tolerance to biotic and abiotic stresses. Further studies are required to enhance the essential traits such as improvement in the resistance to pathogens and abiotic stresses, yield, and nutritional aspects (such as enrichment of lycopene) in tomato and other agronomic crops. With the newly emerging CRISPR/Cas systems, the plant genetic engineering would increase the scope of generating plants with improved flavor, nutrition, and stress tolerance.

## Figures and Tables

**Table 1:** Genome editing of tomato cultivars in response to biotic stress. APETALA2-domain transcription factor *(SlSHN3),* basic helix–loop–helix transcription factor *(SlMYC2),* Chitinase 1 gene from *Helicoverpa armigera (HaCH1),* coat protein *(CP)* or replicase *(Rep) of* TYLCV, *Dicer-like (DCL1),* DNA binding protein from starved cells of *Ralstonia solanacearum (RsDps), Downy mildew resistance 6-1 (SlDMR6-1),* early TYLCV replication associated protein gene *(C1), Fusarium* MAP kinase *(Fmk1), High osmolarity glycerol pathway genes (Hog1* and *Pbs2*), hairpin RNA (hpRNA) construct derived from Potato spindle tuber viroid (PSTVd), *JASMONATE ZIM DOMAIN2 (SlJAZ2), MAP kinase3 (SlMAPK3), MILDEW RESISTANT LOCUS O1 (SlMLO1),* nucleotide-binding site (NBS) and leucine-rich repeat (LRR) gene *(SlNL33),* one of the disrupted genes in reduced mycorrhizal colonization *(rmc)* tomato mutant (Solyc08g075770), *Pectate lyase (SlPL), Powdery Mildew Resistance 4 (PMR4), RESPIRATORY BURST OXIDASE HOMOLOG1 (SlRBOH1),* HD-Zip I family transcription factor (*SlHZ24*), Sucrose non-fermenting 1-related protein kinase 2 *(SnRK2.1* and *SnRK2.2), ToLCV Replicase (Rep),* ToLCV replication initiator protein *(AC4).*

Tomato Cultivar	Target Gene	Effect	Method	Reference
FL8000	*SlDMR6-1*	Mutants showed resistance to *Psuedomonas syringae* pv. Tomato and *Phytophthora capsici*	CRISPR/Cas9	[266]
Micro-Tom	*SlMYC2*	Decreased disease resistance to *B.cinerea*	CRISPR/Cas9	[267]
Micro-Tom	*SlSHN3*	Susceptible to *B. cinerea* infection	RNAi	[242]
Micro-Tom	*SlPL*	Reduced susceptibity to *B. cinerea* infection	RNAi	[243]
Ailsa Craig	*SlNL33*	Increased tolerance to grey mold disease by *B. cinerea* and oxidative stress by methyl viologen	RNAi	[244]
Micro-Tom	*SlLBD40*	Increased tolerance to drought	CRISPR/Cas9	[268]
Moneymaker	*SlMLO1*	Resistant to powdery mildew	CRISPR/Cas9	[249]
Moneymaker	*PMR4*	Resistance against powdery mildew	CRISPR/Cas9	[250,269]
76R	*Solyc08g075770*	Susceptibility to *Fusarium* wilt disease	CRISPR/Cas9	[248]
Ailsa Craig	*MAPK3*	Susceptibility to gray mold disease	CRISPR/Cas9	[247]
Moneymaker	*PMR4*	Resistance against powdery mildew	RNAi	[251]
F, oxysporum f. sp. lycopersici	*MAP kinases (Fmk1, Hog1, Pbs2)*	Resistant to *F. oxysporum*	RNAi	[246]
Ailsa Craig	*SlRBOH1*	Susceptible to root-knot nematode infection	RNAi	[270]
Ailsa Craig	*SlHZ24*	Susceptible to oxidative stress	RNAi	[265]
Pusa Early Dwarf	*HaCH1*	Mutants resistant to pest *Helicoverpa armigera*	RNAi	[271]
Moneymaker	*Cp* and *Rep* of virus	Resistance against TYLCV	CRISPR/Cas9	[252]
M82	*DCL1*	Susceptibility to potato virus X, *Tobacco mosaic virus* (TMV), *Tomato Mosaic virus* (ToMV)	CRISPR/Cas9	[272,273]
Moneymaker	*JAZ2*	Resistance against *banana streak virus*	CRISPR/Cas9	[256]
Tomato	ToLCV *Rep*	Resistant to ToLCV infection	RNAi	[255]
Campbell-28	ToLCV *C1*	Resistant to ToLCV infection	RNAi	[253]
Tomato	ToLCV *AC4*	Resistant to ToLCV infection	RNAi	[254]
Moneymaker	Hairpin RNA (hpPSTVd)	Resistant to Potato spindle tuber viroid infection	RNAi	[274]
Y19	*SlMAPK3*	Susceptible to TYLCV infection	RNAi	[275]

**Table 2:** Genome editing of tomato cultivars in response to abiotic stress tolerance. A putative Na+/H+ antiporter gene (*SlSOS1*), *AGAMOUS-LIKE6 (SlAGL6), Altered response to salt stress 1 (SlARS1), ASCORBATE OXIDASE (SlAO)*, Basic region/Leucine zipper transcription factor (*SlbZIP1*), *BRASSINAZOLE RESISTANT1 (SlBZR1), Chilling-tolerance divergence1 (LeCOLD1), C-repeat binding factor (SlCBF1), Cys2/His2-type zinc-finger protein (SlZF3), DNA-binding with one finger 22 (SlDof22), Fructose 1,6 biphospahte aldolase (SlFBA1), Glutamate decarboxylases (SlGADs), GABA transaminases (SlGABA-Ts), Glutamate receptor-like (SlGLR3.3 and SlGLR3.5), Guanine nucleotide-binding protein alpha-1 subunit (LeGPA1), invertase inhibitor1 (SlINVINH1), JUNGBRUNNEN1 (SlJUB1),* MADS-BOX transcription factor (*SlMBP11*), MADS-BOX transcription factor *(SlMBP8), MAP kinase1 (SlMPK1), MAP kinase3 (SlMAPK3), Mitochondrial Alternate oxidase 1a (SlAOX1a), MIXTA-like MYB transcription factor (SlMX1),* non-expressor of pathogenesis-related gene 1 *(SlNPR1), Phytol kinase (SlVTE5),* Plant specific NAC Transcription factor *(SlNAC11)*, Plant specific Transcription factor *(SlGRAS4),* Proline-,lysine-,glutamic-rich protein gene *(SpPKE1)*, RING-H2 finger gene (ShATL78- Like), *SUMO E3 ligase1 (SlSIZ1),* Tomato 2-oxoglutarate-dependent dioxygenase gene (*SlF3HL*), *Tomato Ethylene response factor genes (TERF2/LeERF2), Tomato osmotic sensitive-1 (tos1), Tomato salt sensitive-2 (tss2), uridine diphosphate glucosyltransferase (SlUGT75C1), Whirly1 (SlWHY1).*

Tomato Cultivar	Target Gene	Effect	Method	Reference
Ailsa Craig	*SlCBF1*	Reduced chilling tolerance	CRISPR/Cas9	[312]
Lichun	*TERF2/LeERF2*	Susceptible to freezing	RNAi	[279]
XF-2	*SlINVINH1*	Increased tolerance to chilling stress	RNAi	[284]
*Solanum lycopersicum*	*SlFBA*	Decreased tolerance to chilling stress	RNAi	[288]
Ailsa Craig	*SlF3HL*	Enhanced sensitivity to chilling stress	RNAi	[313]
Yaxin 87-5	*LeCOLD1*	Susceptible to chilling stress	RNAi	[314]
Zhong-shu 6	*SlWHY1*	Susceptible to chilling stress	RNAi	[315,316]
Micro-Tom	*SlGRAS4*	Susceptible to chilling stress	RNAi	[317]
Ailsa Craig	*SlGLR3.3 and SlGLR3.5*	Susceptible to chilling stress	VIGS	[318]
Yaxin 87-5	*LeGPA1*	Susceptible to cold stress	RNAi	[319]
Ailsa Craig	*SlMAPK3*	SlMAPK3 is induced by drought stress	CRISPR/Cas9	[320]
Ailsa Craig	*SlNPR1*	The mutants exhibited reduced drought tolerance	CRISPR/Cas9	[321]
Zhongshu No.5 (ZS5)	*SlAO*	Increased photosynthetic activity under drought stress	RNAi	[322]
Ailsa Craig	*SlMX1*	Susceptible to drought and reduced carotenoid levels in fruits	RNAi	[323]
*S. habrochaites* (LA1777)	*ShATL78- Like*	Susceptible to cold and drought	RNAi	[324]
Micro-Tom	*SlUGT75C1*	Increased tolerance to drought stress	RNAi	[300]
Ailsa Craig	*SlMBP8*	Enhanced tolerance to drought and salt stress	RNAi	[305]
Micro-Tom	*SlPP2C1*	Resistant to drought stress	RNAi	[325]
Micro-Tom	*SlAOX1a*	Susceptible to drought stress	RNAi	[326]
M82	*SpPKE1*	Susceptible to drought stress	RNAi	[327]
Moneymaker	*SlJUB1*	Susceptible to drought stress	VIGS	[328]
Condine Red	*SlBZR1*	The heat stress tolerance was reduced in the mutant	CRISPR/Cas9	[298]
M82	*SlAGL6*	The mutants produce fruits under heat	EMS mutagenesis	[329]
Moneymaker	*SlHsfA1*	Sensitive to heat stress	RNAi	[293]
Micro-Tom	*SlVTE5*	Sensitive to high light and high temperature	RNAi	[330]
OFSN	*SlMPK1*	Enhanced tolerance to heat	RNAi	[292]
M82	*SlSIZ1*	Reduced tolerance to heat stress	RNAi	[331]
M82	*SlWHY1*	Susceptible to heat stress	RNAi	[332]
Moneymaker	*tos1*, and *tss2*	The mutants are hypersensitive to osmotic stress	EMS mutagenesis	[311]
Micro-Tom	*SlSnRK2.1, SlSnRK2.2*	Enhanced tolerance to osmotic stress	RNAi	[310]
Pera	*SlSOS1*	Increased sensitivity to salinity	RNAi	[333]
Ailsa Craig	*SlDof22*	Decreased tolerance to salinity	RNAi	[334]
Ailsa Craig	*SlMBP11*	Decreased sensitive to salt stress	RNAi	[304]
Ailsa Craig	*SlNAC11*	Susceptible to salinity and drought stress	RNAi	[335]
Ailsa Craig	*SlZF3*	Susceptible to salt stress	RNAi	[306]
Ailsa Craig	*SlbZIP1*	Reduced tolerance to salinity and drought stresses	RNAi	[303]
Moneymaker	*SlARS1*	Decreased transpiration water loss under salt stress	Transposon DNA	[336]
Lichun	*SlGADs, SlGABA-Ts*	Susceptible to salt stress	VIGS	[307]

**Table 3:** Genome editing of tomato cultivars in response to combined biotic and abiotic stresses. Abscisic acid-induced MYB1 (SlAIM1), Hybrid proline-rich protein1 (HyPRP1), Stress-related NAC1 (SlSRN1), Tomato ethylene response factor (SlPti4), Tomato lipoxygenase gene (SlTomLoxD), Tomato SR/CAMTA transcription factors (SlSR1 and SlSR3L).

Tomato Cultivar	Target Gene	Effect	Method	Reference
CastlemartII, Micro-Tom	*SlAIM1*	Increased susceptibility to *B. cinerea* and increased sensitivity to salinity and oxidative stress	RNAi	[341]
Suhong 2003	*SlSRN1*	Positive regulator of defense responses against *B. cinerea* and Pseudomonas Negative regulator of drought and oxidative responses	VIGS	[337]
Suhong 2003	*SlSR1, SlSR3L*	Enhanced resistance to *B. cinerea* and susceptible to drought stress	VIGS	[337]
Micro-Tom	*SlPti4*	Susceptible to drought and weak resistant to *B. cinerea* infection	RNAi	[362]
M82 and *S. pennellii*	*HyPRP1*	Increased tolerance to salinity and oxidative stress	RNAi	[358]

**Table 4:** List of gene editing for yield and fruit quality improvement in tomato. AGAMOUS-LIKE6 (SlAGL6), Alcobaca (ALC), Anthocycanin1 (ANT1), ARGONAUTE7 (AGO7), Aux/IAA9 transcription factor (SlIAA9), BLADE-ON-PETIOLE family (BOP1, BOP2, BOP3), mitochondrial transcription factor A (TFAM1, TFAM2), Carotenoid isomerase (CRTISO), CLAVATA3 (CLV3), Defective chloroplasts and leaves (DCL), DELLA (aspartic acid–glutamic acid–leucine–leucine–alanine), fasciated and branched (fab) and fasciated inflorescence (fin), FEEBLY (FB), GDP-L-GALACTOSE PHOSPHORYLASE (GGP1), LEAFY-COTYLEDON1-LIKE4 (L1L4), LYCOPENE BETA CYCLASE (CycB), MULTIFLORA (MULT), organelle RNA recognition motif-containing protein4 (ORRM4), OVATE (O), Phytoene desaturase (PDS), pyruvate-dependent GABA-T family proteins (GABA-TP1, GABA-TP2, GABA-TP3), cationic amino acid transporter 9 (CAT9), succinate semialdehyde dehydrogenase (SSADH), RIPENING INHIBITOR (RIN), SELF PRUNING (SP), SELF PRUNING 5G (SP5G), SHORT ROOT (SHR), stay-green 1 (SGR1), lycopene ε-cyclase (LCY-E), beta-lycopene cyclase (Blc), lycopene b-cyclase 1 (LCY-B2), WUSCHEL (WUS).

Tomato Cultivar	Target Gene	Effect	Method	Reference
*Solanum pimpinellifolium*	*fab* and *fin*	Branched inflorescences with fasciated flowers, an increase in fruit size	EMS and fast neutron (FN)	[363]
M82	Homolog of Arabidopsis *S* and *SP*	Compound inflorescence	CRISPR/Cas9	[364]
*M82*	*SP5G*	Early flowering	CRISPR/Cas9	[365]
*S. pimpinellifolium*	*SP*	Determinate growth habit	CRISPR/Cas9	[366]
*S. pimpinellifolium*	*SP and SP5G*	Compact tomato plants, synchronous fruit ripening	CRISPR/Cas9	[366]
*S. pimpinellifolium*	*SP*	Determinate growth habit	CRISPR/Cas9	[367]
*S. pimpinellifolium*	*MULT*	Increased fruit number	CRISPR/Cas9	[367]
M82	*CLV3*	Increased locule number and fruit weight	CRISPR/Cas9	[364]
*S. pimpinellifolium*	*CLV3*	Increased locule number and fruit weight	CRISPR/Cas9	[367]
*S. pimpinellifolium*	*WUS*	Enlarged fruit size	CRISPR/Cas9	[366]
*S. pimpinellifolium*	*O*	Fruit shape	CRISPR/Cas9	[367]
Ailsa craig	*RIPENING INHIBITOR* (*RIN*)	Fruit ripening	CRISPR/Cas9	[368,369,370]
Micro-Tom	*ORRM4*	Fruit ripening	CRISPR/Cas9	[371]
M82	*ALC*	Long shelf life	CRISPR/Cas9	[372]
Pusa Ruby	*SlGH3.2*	Increased shelf life	RNAi	[373]
TYLCV tolerant line MP-1	*AGL6*	Parthenocarpy	CRISPR/Cas9	[329]
Micro-Tom and Ailsa craig	*IAA9*	Parthenocarpy, leaf shape	CRISPR/Cas9	[374]
*Yellow flesh e^3756^*, *Bicolor^cc383^*, M82 and *S. pimpinellifolium^LA1578^*	*PSY1*	Yellow fruits	CRISPR/Cas9	[385]
*S. pimpinellifolium*	*CycB*	Lycopene enriched	CRISPR/Cas9	[367]
Ailsa craig	*SGR1, LCY-E, Blc, LCY-B2*	Lycopene enriched	CRISPR/Cas9	[375]
M82 and *S. pennellii* IL 10-2	*CRTISO* (*Tangerine*)	Orange fruits	Fast neutrons	[14]
Micro-Tom	*MYB (ANT1)*	High anthocyanin	TALENs, CRISPR/Cas9	[57]
Moneymaker	*FB*	High anthocyanin	Transposon	[181]
Heinz 1706	*L1L4*	Fruit metabolites	ZFN	[376,377]
Ailsa craig and Micro-Tom	*GABA-TP1, GABA-TP2, GABA-TP3, CAT9, SSADH*	Enhanced GABA content in leaves	CRISPR/Cas9	[378]
Ailsa craig and Micro-Tom	*GDP-L-GALACTOSE PHOSPHORYLASE (GGP1)*	Increased foliar ascorbic acid content	CRISPR/Cas9	[378]
M82	*AGO7*	Change in leaf shape	CRISPR/Cas9	[61]
M82	*BOP1, BOP2, BOP3, TFAM1, TFAM2*	Altered leaf complexity and loss of floral organ abscission, fused floral organs, defects in fruit shape	CRISPR/Cas9	[380]
Micro-Tom	*DELLA*	Reduced serrated leaflets	CRISPR/Cas9	[379]
Micro-Tom	*PDS*	Albino	CRISPR/Cas9	[381]
*S. pimpinellifolium*	*PDS*	Albino	CRISPR/Cas9	[366]
Moneymaker	*DCL*	Albino leaves with green sectors	Transposon	[382]
*Tomato* spp.	*SHR*	Short root	CRISPR/Cas9	[383]
M82	*PROCERA*	Enhanced GA response	TALENs	[384]

## Data Availability

Not applicable.

## Abbreviations

*AC4*	ToLCV replication initiator protein
*AGO7*	*ARGONAUTE7*
*ALC*	*Alcobaca*
*ANT1*	*Anthocycanin1*
*Blc*	*beta-lycopene cyclase*
*BOP1, BOP2, BOP3*	*BLADE-ON-PETIOLE* family
*C1*	early TYLCV replication associated protein gene
*CAT9*	*cationic amino acid transporter 9*
*CLV3*	*CLAVATA3*
CMTs	chromomethylases
CNR	colourless non-ripening
*CP* and *Rep*	coat protein and Replicase of TYLCV
Cpf1	CRISPR-associated endonuclease in Prevotella and Francisella 1
CRISPR/Cas9	Clustered Regularly Interspaced Short Palindromic Repeat/CRISPR- associated protein 9)
*CRTISO*	*Carotenoid isomerase*
*CycB*	*LYCOPENE BETA CYCLASE*
*DCL*	*Defective chloroplasts and leaves*
*DCL1*	*Dicer-like*
DELLA	aspartic acid–glutamic acid–leucine–leucine–alanine
DES	Diethyl sulfate
DHFR	*Dihydroflavonol* 4-reductase
DRM	Domains rearranged methyltransferases
DSB	Double-stranded DNA break
dsDNA	Double stranded DNA
EI	ethyleneimine
EMS	ethyl methanesulphonate
ENU	ethyl nitro urethane
ENU	ethyl nitrosourea
*fab*	*fasciated and branched*
FAO	Food and Agricultural Organization
*FB*	*FEEBLY*
*fin*	*fasciated inflorescence*
*Fmk1*	*Fusarium* MAP kinase
*GABA-TP1, GABA-TP2, GABA-TP3*	*pyruvate-dependent GABA-T family proteins*
GE	Genome editing
*GGP1*	*GDP-L-GALACTOSE PHOSPHORYLASE*
GMOs	Genetically modified organisms
gRNA	Guide RNA
GWAS	Genome Wide Association study
*HaCH1*	Chitinase 1 gene from *Helicoverpa armigera*
HDR	Homology-dependent recombination
*Hog1* and *Pbs2*	*High osmolarity glycerol pathway genes*
hpRNA	hairpin RNA
*HyPRP1*	*Hybrid proline-rich protein1*
INDELs	insertions or deletions
*L1L4*	*LEAFY-COTYLEDON1-LIKE4*
*LCY-B2*	*lycopene b-cyclase 1*
*LCY-E*	*lycopene ε-cyclase*
*LeCOLD1*	*Chilling-tolerance divergence1*
*LeGPA1*	*Guanine nucleotide-binding protein alpha-1 subunit*
MET1	Methyltransferase1
MNU	1-methyl-1-nitrosourea
*MULT*	*MULTIFLORA*
NGS	Next-generation sequencing
NHEJ	Non-homologous end joining
*O*	*OVATE*
ODM	Oligonucleotide directed mutagenesis
*ORRM4*	*organelle RNA recognition motif-containing protein4*
PAM	protospacer adjacent motif
*PDS*	*Phytoene desaturase*
*PMR4*	*Powdery Mildew Resistance 4*
PSTVd	Potato spindle tuber viroid
PTGS	post-transcriptional gene silencing
PVX	Potato virus X
QTL	Quantitative trait loci
RdDM	RNA-dependent DNA methylation
RdRP	RNA dependent RNA polymerase
*Rep*	Replicase of TOLCV
*RIN*	*RIPENING INHIBITOR*
RISC	RNA-induced silencing complex
*rmc*	reduced mycorrhizal colonization
RNA pol IV	RNA polymerase IV
RNA pol V	RNA polymerase V
RNAi	RNA interference
*RsDps*	DNA binding protein from starved cells of *Ralstonia solanacearum*
RVD	Repeat Variable Diresidue
*SGR1*	*stay-green 1*
ShATL78- Like	RING-H2 finger gene
*SHR*	*SHORT ROOT*
siRNAs	short interfering RNAs
*SlAGL6*	*AGAMOUS-LIKE6*
*SlAIM1*	*Abscisic acid-induced MYB1*
SlAlkBH2	AlkB homolog 2
*SlAO*	*ASCORBATE OXIDASE*
*SlAOX1a*	*Mitochondrial Alternate oxidase 1a*
*SlARS1*	*Altered response to salt stress 1*
*SlbZIP1*	Basic region/Leucine zipper transcription factor
*SlBZR1*	*BRASSINAZOLE RESISTANT1*
*SlCBF1*	*C-repeat binding factor*
*SlDML2*	Domains rearranged methyltransferases
*SlDMR6-1*	*Downy mildew resistance 6-1*
*SlDof22*	*DNA-binding with one finger 22*
*SlF3HL*	Tomato 2-oxoglutarate-dependent dioxygenase gene
*SlFBA1*	*Fructose 1,6 biphospahte aldolase*
*SlGABA-Ts*	*GABA transaminases*
*SlGADs*	*Glutamate decarboxylases*
*SlGLR3.3*	*Glutamate receptor-like*
*SlGLR3.5*	*Glutamate receptor-like*
*SlGRAS4*	Plant specific Transcription factor
*SlHZ24*	HD-Zip I family transcription factor
*SlIAA9*	*Aux/IAA9 transcription factor*
*SlINVINH1*	*invertase inhibitor1*
*SlJAZ2*	*JASMONATE ZIM DOMAIN2*
*SlJUB1*	*JUNGBRUNNEN1*
*SlMAPK3*	*MAP kinase3*
*SlMAPK3*	*MAP kinase3*
*SlMBP11*	MADS-BOX transcription factor
*SlMBP8*	MADS-BOX transcription factor
*SlMLO1*	*MILDEW RESISTANT LOCUS O1*
*SlMPK1*	*MAP kinase1*
*SlMX1*	*MIXTA-like MYB transcription factor*
*SlMYC2*	basic helix–loop–helix transcription factor
*SlNAC11*	Plant specific NAC Transcription factor
*SlNL33*	nucleotide-binding site (NBS) and leucine-rich repeat (LRR) gene
*SlNPR1*	non-expressor of pathogenesis-related gene 1
*SlPL*	*Pectate lyase*
*SlPti4*	*Tomato ethylene response factor*
*SlRBOH1*	*RESPIRATORY BURST OXIDASE HOMOLOG1*
*SlSHN3*	APETALA2-domain transcription factor
*SlSIZ1*	*SUMO E3 ligase1*
*SlSOS1*	A putative Na+/H+ antiporter gene
*SlSR1*	*Tomato SR/CAMTA transcription factors*
*SlSR3L*	*Tomato SR/CAMTA transcription factors*
*SlSRN1*	*Stress-related NAC1*
*SlTomLoxD*	*Tomato lipoxygenase gene*
*SlUGT75C1*	*uridine diphosphate glucosyltransferase*
*SlVTE5*	*Phytol kinase*
*SlWHY1*	*Whirly1*
*SlZF3*	*Cys2/His2-type zinc-finger protein*
*SnRK2.1*	Sucrose non-fermenting 1-related protein kinase 2
*SnRK2.2*	Sucrose non-fermenting 1-related protein kinase 2
*SP*	*SELF PRUNING*
*SP5G*	*SELF PRUNING 5G*
*SPB3-like*	*SQUAMOSA promoter binding protein3-like*
*SpPKE1*	Proline-,lysine-,glutamic-rich protein gene
*SSADH*	*succinate semialdehyde dehydrogenase*
TALENs	Transcriptional Activator Like Effector Nucleases
T-DNA	Transposon DNA
TE	Transposable elements
*TERF2/LeERF2*	*Tomato Ethylene response factor genes*
*TFAM1, TFAM2*	mitochondrial transcription factor A
TGS	transcriptional gene silencing
TILLING	Targeted Induced Local Lesions IN Genomes
TMV	Tobacco Mosaic Virus
*tos1*	*Tomato osmotic sensitive-1*
TRV	Tobacco Rattle Virus
*tss2*	*Tomato salt sensitive-2*
TYMV	Turnip yellow Mosaic Virus
VIGS	Virus-induced gene silencing
*WUS*	*WUSCHEL*
ZFNs	Zinc finger nucleases
